# Young Adults and Sick Leave Length of Mental Illnesses

**DOI:** 10.3389/fpubh.2022.882707

**Published:** 2022-06-13

**Authors:** Beata Gavurova, Miriama Tarhanicova

**Affiliations:** ^1^Faculty of Management and Economics, Tomas Bata University in Zlín, Zlín, Czechia; ^2^Faculty of Mining, Ecology, Process Control and Geotechnologies, Technical University of Košice, Košice, Slovakia

**Keywords:** mental diseases, substance use, health literacy, young adults, public health

## Abstract

**Objectives:**

The objective was to explore whether a sick leave length related to mental morbidity differs across different occupational categories.

**Methods:**

In the analysis, registry of sick leaves was analyzed. Provided analysis is focused on the length of sick leaves related to mental diseases caused by substance use or other factors. Dependent variable is the sick leave length, and the independent variables are the categories of disease and occupation. Kruskal–Wallis test, Shapiro–Wilk test, and Brown–Forsythe (B–F) are used.

**Results:**

There are differences in mental sick leave lengths caused by substance use or other factors. In the case of mental illnesses attributable to drugs, differences in the sick leave duration among different working groups were not found. Considering mental disorders caused by other factors, there are differences in the sick leave duration among different working groups.

**Conclusions:**

There is no evidence of longer sick leave in people diagnosed with mental disorder related to substance use. Differences in occupational categories do not relate to sick leave length.

## Introduction

Nowadays, countries with rapidly aging populations and low birth rates face the challenge of growing economic burden of the future generations. This challenge attracts the attention of policymakers as well as the research teams across the world to examine the health and socioeconomic conditions and the ability of young adults to bring economic value to country. More attention is paid to mental health as young people are daily exposed to stressful life events that negatively affect their health. Stressful events may be the consequence of low social status, parental and peer relationship quality, social exclusion, etc. (1). People diagnosed with chronic stress or exposed to any other life event are endangered more by mental diseases (2).

Mental health is important in young people because of the development of cognitive, social, and emotional being of young people as well. According to Birn et al., people exposed to high level of stress in early childhood are at higher risk of a wide range of behavioral problems or mental disorders (3). Global Burden Disease Study 2017 (GBD) shows that mental disorders have consistently formed more than 14% of age-standardized years lived with disability (YLDs) for nearly three decades. Mental diseases have >10% prevalence in all 21 GBD regions (4). Severe mental illness is difficult to treat, and thus, enormous pharmacological and psychological resources are needed to intervene. Treatment and prevention of mental disorders require the interaction of various social and biological risk and protective factors throughout the life course (5). As stated by Mather et al., mental disorders are a major public health problem worldwide and the leading cause of work disability (sick leave and disability pension) in Europe. Generally, sick leave becomes more common with age; however, sick leave due to mental disorders is the most common in a younger age. Sick leave due to mental disorders has been found to have a negative effect on future work life, such as a higher risk of unemployment and a lower income (6). Salomonsson et al. confirm that sick leave due to common mental disorders (CMDs) increases rapidly and presents a major societal challenge (7). Mather et al. emphasize that sick leave due to mental disorders increased the risk of reoccurring sick leave within 2 years, disability pension and unemployment, independent of genetics, and shared environment (8). Lidwall et al. stress out that the risk of sick leave with mental disorder is higher among women compared to men, among persons aged 30–39 and among parents in families with underage children (9).

Substance use presents high risk of development of mental disorders. Children and adolescents affected by alcohol use might have adverse health effects related to mental disorders. Lima et al. highlight a bidirectional association between alcohol use and mental health condition, where the presence of one issue almost doubles the risk of having the other issue (10). According to Amiri and Behnezdad, it can be concluded that alcohol consumption increases the risk of sick leave in working population (11). In the study of Malla et al., the authors emphasize the early onset of most mental disorders and substance abuse as well as their persistence into adulthood. Moreover, Malla et al. warn about the delays in seeking professional help, which negatively affects not only young people but also society as well. There is a necessity of different conceptual frameworks for the treatment of youth mental disorders and transformation of provided services. It is necessary to not only reduce the unmet needs but also allow a more meaningful exploration of the nature of such problems and the best way to treat them (12). Inability to recognize mental health problems of younger people hinders early detection and help-seeking behaviors (13).

The way people treat themselves is highly influenced by their health literacy. According to Liu, health literacy is an important determinant of health (14). Health literacy is a function of many factors, mostly social and individual. Wittink and Oosterhaven state that health literacy is linked to literacy and entails people's knowledge, motivation, and competences to access, understand, appraise, and apply health information to make judgements and take decisions in everyday life concerning health care, disease prevention, and health promotion to maintain or improve quality of life during the life course (15).

According to Fleary et al., there is a meaningful relationship between health literacy and adolescents' health behaviors (16). Duplaga's research on health literacy, which took place in Poland, showed that lower health literacy is associated with a lower self-assessment of health, the prevalence of obesity and disability, less frequent physical activity, lower consumption of fruits and vegetables, and with more frequent hospitalizations (17). Carod-Artal informs on the factors associated with poor mental health such as poverty, low educational level, gender discrimination, unhealthy lifestyle, violence, physical ill-health, unemployment, social exclusion, and human rights violations (18, 19). Broder et al. admit that the unique particularities of children and young people relevant for health literacy include their disease and health-risk profiles, their vulnerability to demographic factors, their social role and status, and their right to participation (20).

There are also gender differences in mental health of people. Mental disorders affect more deeply women compared to men at every level of household income (18). More vulnerable groups of people are young people with lower education. Such people occupy lower job positions at work, which are not demanding on the attained educational level, such as elementary occupations, plant and machine operators and assemblers, craft and related trade workers, skilled agricultural, forestry, and fishery workers. Moreover, Milner et al. state that improving employment opportunities could reduce nearly half of the adverse effects of low education on mental health of young people (21).

More effective interventions would be reached if there were closer links between health literacy and behavior change frameworks (22). Therefore, it is necessary to increase the health literacy in younger age. Sukys et al. warn that health literacy among young university students is insufficient (23). Individuals must understand and use information regarding how to benefit from healthcare services and maintain a healthy life. The increase in health literacy might increase the participation of individuals in promoting healthy behaviors. All behaviors that can affect health must be under control (24). Mental health literacy can be increased with specific interventions, which provided in the primary care or in mainstream or online media (13, 25, 26).

Low levels of health literacy might be a problem in work environment, as people with lower health literacy are at higher risk of spreading the illness and of longer period needed for treatment (15, 17, 19, 22). Longer treatment expressed by a longer sick leave duration represents a serious economic burden not only to company, but also, in the form of lost productivity, to society as well. Sick leave, associated with mental illness as well as any other types of illness, represents long-term expenditures for the state, especially in economies where the healthcare system is financed from the state money. Young people who have a mental illness or disorder are not only current economic burden, but also a future one. The sick leave duration varies depending on the type of disease. Some diseases are treatable whereas others are incurable or have lasting consequences that results into financial dependence on family members or state aid. These facts were the motivation to our research aiming to find out whether the occupational types of young people affect the length of their incapacity for work due to mental illnesses acquired through the use of addictive substances or other risk factors.

In this study, sick leave length is compared in regard to the types of mental disease and the work/occupational categories in the case of the Czech Republic. Mental diseases are divided into two groups: mental diseases related to substance use (legal and illegal drugs) and mental diseases related to other factors (dementia, behavioral syndromes associated with physiological disturbances and physical factor, behavioral and emotional disorders, etc.). The difference in the length of sick leave between occupational categories is supposed, as those categories are differentiated by the level of health literacy. Limited health literacy influences how people care about their health and their health prevention. The differences in the length of sick leave are expected in the case of mental illnesses caused by addictions, as addicted people are more likely to be in working categories that does not need any further level of education.

## Methods

This study aims to examine the differences in the length of sick leaves related to alcoholic and non-alcoholic mental disorders and diseases, which is defined according to International Statistical Classification of Diseases and Related Health Problems (ICD-10) as diagnoses F00–F99 (27). In the analysis, database of sick leaves from 2017, covering the sick leaves in the Czech Republic, was used. This database includes data on sick leave starting from first day of working absence to last day of sick leave. In the Czech Republic, persons participating in the sickness insurance are entitled to sickness cash benefit (dávka v nemoci or nemocenské) when they are temporarily incapable to work or in quarantine starting from the 14th day of their sick leave. People who are eligible for sickness benefits are those who are employed or self-employed or at least, the ones who pay sickness insurance contribution. If monthly income is higher or equals to EUR 117.11, it is compulsory by law to participate in sickness insurance. Sickness benefit is given by employer from first to 14th day of sick leave; if the sick leave is longer than 14 days, then sickness benefit is given to employee by sickness insurance. Self- employed people are given sickness benefit starting from 15th day of their sickness. There are the limits based on which the sickness benefit is calculated. Sickness benefit is calculated as 60% of daily income for the first 30 days of sickness; 66% of daily income for the next 30 days; and 72% of daily income for the days that exceeds 61.

In our analysis, we consider all period of sick leave (from starting day to the last day). The diagnose issued on patient's transcription is determined based on the examination of mental health specialist. Analyzed data were provided by the Czech Social Security Administration as a part of research project A-86-19: “Economic Quantification of Social Costs of the Use of Alcohol, Tobacco and Illicit Drugs in the Czech Republic,” approved by the Ethical Committee of the General University Hospital in Prague. The main aim of this project was to quantify the social costs related to drug abuse. To further analyze the substance use problem in the Czech Republic, in this article, we distinguish between sick leave length related to substance use and related to other causes.

As mental diseases are dangerous mostly in younger people, because young people present potential future economic burden, we analyzed only sick leaves of people aged up to 29 years (6). Eurostat defines young adults as people aged 15–29 years (28). Database of sick leave provides detailed information about each case of sick leave such as demographic factors, diagnosis related to sick leave, and category of patients' work. In this database, there are only sick leave covering employees, self-employed people, and people paying the social insurance, and data on unemployed people are missing.

Every case of sick leave is therefore defined by patient's:

- age—calculated as the difference between year 2017 and the year of birth,
- sex—male, female,
- diagnosis—corresponding code of diagnosis (according to ICD−10); it is the diagnosis that relate to sick leave,
- length of sick leave—calculated as the difference between the end date and start date of sick leave,
- occupations category—there are 10 working categories (according to work classification CZ–ISCO),
- ID—numerical code,
- NUTS 3 of employer—NUTS 3 code of place of work,
- NUTS 3 of doctor—NUTS 3 code of place of doctor,
- NUTS 3 of patient—NUTS 3 code of place where patient lives.

There are 10 work categories defined by the CZ-ISCO classification of occupations (29). First working category presents managers. That position usually requires higher education. On the contrary, the last (the ninth) category presents elementary occupations. All occupational categories are presented in Table 1.

**Table 1:** The international standard classification of occupations (31).

**N**°****	**Work category**
0	Armed forces occupations
1	Managers
2	Professionals
3	Technicians and associate professionals
4	Clerical support workers
5	Services and sales workers
6	Skilled agricultural, forestry and fishery workers
7	Craft and related trades workers
8	Plant and machine operators and assemblers
9	Elementary occupations

It is supposed that the work category hardly relates to education, whereas the level of education attained to a large extent affects employability and the type of work performed. Next, compared to people with lower education, it is supposed that people with higher education will take care of their health more with emphasis on better prevention. In general, health literacy is higher in people with higher education. On the contrary, people with lower education might have more social problems related to addiction or any kind of psychical issue, etc. Moreover, as lower educated people are supposed to have lower health literacy, their sick leave might be longer; therefore, this study aims to determine the differences in the length of sick leave across different work types as follows:

- differences in the length of sick leave within different mental diagnose group (groups F10–F19 to groups F00–F09, F20–F99).
- differences in the length of time related to sick leave attributable to diagnoses defined as F10–F19 within different work categories.
- differences in the sick leave length attributable to diagnoses defined as F00–F09, F20–F99 within different work categories.
- pairwise differences in case of sick leave related to F00–F09, F20–F99.

In general, to examine this kind of relationship, the ANOVA, analysis of variance, is used. However, according to Shapiro–Wilk test, the assumption of normality was violated. Therefore, Kruskal–Wallis and independentsamples Mann–Whitney U test were performed to examine the existence of differences in the duration of sick leave as both are a non-parametric alternatives to ANOVA (30, 31). The duration of sick leave, considered as dependent variable, is not from the normal distribution, since the majority of sick leaves took shorter period of time. Therefore, the distribution is skewed to the right. Brown–Forsythe (B–F) was used to test an assumption of equal variances that was not violated.

According to our assumptions, the tested hypotheses are as follows:

- H1: There is the difference in the length of sick leave comparing mental diagnoses caused by substances (diagnoses F10–F19 according to ICD−10) and mental diagnoses caused by other issues (F00–F09, F20–F99 according to ICD−10).
- H2: Comparing different working categories, there is the difference in median of length of sick leaves related to substance use (F10–F19).
- H3: Comparing different working categories, there is the difference in median of length of sick leaves related to other issues (F00–F09, F20–F99).
- H4: Sample 1 and Sample 2 distributions are the same.

The design and realization of the study were approved by the ethics committee of the General University Hospital in Prague as individual research (ref. 915/20 S–IV).

## Results

First, we divided all cases of sick leave related to mental disorder into two groups, and then, we examined the differences within both groups by using independentsamples Mann–Whitney U test. The Mann–Whitney U test was selected as the dependent variable (length of sick leave) is not from normal distribution, and we compare only two groups. Those groups are as follows:

- mental disorders/diagnoses related to the consumption of substance (alcohol, tobacco, illegal drugs), therefore the diagnoses—defined by ICD−10 as F10–F19;
- mental disorders/diagnoses related to other causes—defined by ICD−10 as F00–F09 and F20–F99.

The first part of analytical processes consists of determining descriptive statistics for the variable length of sick leave, presented in Table 1 in relation to first hypothesis:

*H1: There is a difference in the length of sick leave comparing mental diagnoses caused by substances (diagnoses F10*–*F19 according to ICD*–*10) and mental diagnoses caused by other issues (F00–F09, F20–F99 according to ICD*–*10)*.

The *p*-value was estimated at 0.0001; therefore, we reject the null hypothesis in favor of the alternative hypothesis at the 5% significance level. According to *p*-value, there are the differences in distribution of the length between mental diagnoses attributable to substance use (F10–F19) and other types of mental diseases (F00–F09, F20–F99). As stated in Table 2, length of sick leave related to mental disease F10–F19 differs from the length of sick leave related to mental disease F00–F09, F20–F99.

**Table 2:** Independent-samples Mann–Whitney U test Summary: the length of sick leave related to mental disorder caused by drugs (alcohol, tobacco, illicit drugs) and other conditions, in the study sample of young adults aged 15–29 years, the Czech Republic, 2017.

**Type**	**Value**
Total *N*	5,981
Mann-Whitney U	1015174,000
Wilcoxon W	16508135,000
Test statistic	1015174,000
Standard error	33925,690
Standardized test statistic	−4,120
Asymptotic Sig. (2-sided test)	<0.001

Mental disorders category: F10–F19 (*N* = 415) has a larger mean rank 3, 327, 8 than mental disorder category related to other causes (*N* = 5 566) with mean rank (2 965,89) and thus tends to take larger values. A statistically significant difference was found (U = 1,015,174, *p* < 0.001).

Next, we performed Kruskal–Wallis H test to test the second hypothesis:

*H2: Comparing different working categories, there is a difference in the median of length of sick leaves related to substance use (F10–F19)*.

As presented in Table 3, the *p*-value was estimated at 0.3136; therefore, we do not reject the null hypothesis in favor of the alternative hypothesis at the 5% significance level. According to *p*-value, there are no differences in the median of the length between separate working categories.

**Table 3:** Kruskal–Wallis test results: the length of sick leave related to mental illnesses caused by drugs (alcohol, tobacco, illicit drugs), in the study sample of young adults aged 15–29 years, the Czech Republic, 2017.

	**Work category**	***N***	**Rank sum**	**Mean rank**
F10–F19: the length of sick leave by work categories	0	12	3258.5	271.54
	1	5	1173.5	234.70
	2	5	1168.5	233.70
	3	26	4941.0	190.04
	4	20	4067.0	203.35
	5	91	19626.0	215.67
	6	5	589.5	117.90
	7	167	35652.5	213.49
	8	35	6884.5	196.70
	9	49	8959.0	182.84
Kruskal–Wallis test details	H statistic	10.47		
	X^2^ approximation	10.47		
	DF	9		
	*p*-value	0.3136		

As opposed to our assumption H2, in the case of mental illnesses attributable to excessive use of alcohol, illegal drugs, or smoking, there are no differences in the duration of sick leave. Our assumption of a longer duration of sick leave in case of people with lower level of education has not been confirmed.

*H3: Comparing different working categories, there is a difference in median of length of sick leaves related to other issues (F00–F09, F20–F99)*.

According to results in Table 4, the *p*-value was estimated at 0.0101; therefore, we reject the null hypothesis in favor of the alternative hypothesis at the 5% significance level. According to *p*-value, there are the differences in median of the length between mental diagnoses attributable to other issues (F00–F09, F20–F99).

**Table 4:** Kruskal–Wallis test results: the length of sick leave related to mental illnesses caused by other conditions, in the study sample of young adults aged 15–29 years, the Czech Republic, 2017.

	**Work category**	***N***	**Rank sum**	**Mean rank**
F00–F09, F20–F99: the length of sick leave (in days) by work categories	0	397	1052902.0	2652.15
	1	79	252820.0	3200.25
	2	80	216649.0	2708.11
	3	562	1582469.5	2815.78
	4	555	1588038.0	2861.33
	5	1,327	3780646.5	2849.02
	6	37	96767.0	2615.32
	7	1,556	4350593.0	2796.01
	8	455	1168787.0	2568.76
	9	518	1403289.0	2709.05
Kruskal-Wallis test details	H statistic	21.62		
	X^2^ approximation	21.62		
	DF	9		
	*p*-value	0.0101		

In the case of mental illnesses F00–F09, F20–F99, we found the differences in the length of sick leave within different work categories. As there are differences within the type of mental disease (related to substance use or not related to substance use) and differences among occupational groups, we compared the average length of sick leave between men and women in regard to ICD−10 groups, shown in Table 5. Therefore, Table 5 shows the average length of sick leave in regard to mental disease in regard to ICD−10 classification and gender. As it may be seen, in the case of men, the greater average length of sick leave is related to intellectual disability (group F70–F79), amounted at 85.63. On the contrary, behavioral syndromes associated with physiological disturbances and physical factors are related to sick leave with the shortest average duration amounted at 20.17 days on average. In the case of women, the longest average sick leave is related to organic, including symptomatic, mental disorders (F00–F09) amounted at 53.11 days on average. Neurotic, stress-related, and somatoform disorders (F40–F49), amounted at 30.89 days on average, are related to shortest duration of sick leave.

**Table 5:** Descriptive statistics: the average length of sick leave in the study sample of young adults aged 15–29 years, according to International Statistical Classification of Diseases and Related Health Problems, version 10, the Czech Republic, 2017.

**ICD−10**	**ICD−10 description**	**Average length of sick leave**	
		**Men**	**Women**
F40–F49	Neurotic, stress-related and somatoform disorders	26.87	30.89
F30–F39	Mood [affective] disorders	35.32	37.37
F20–F29	Schizophrenia, schizotypal and delusional disorders	68.30	52.59
F60–F69	Disorders of adult personality and behavior	44.64	51.38
F10–F19	**Mental and behavioral disorders due to psychoactive substance use**	**44.86**	**43.30**
F50–F59	Behavioral syndromes associated with physiological disturbances and physical factors	20.17	44.57
F00–F09	Organic, including symptomatic, mental disorders	36.79	53.11
F70–F79	Intellectual disability	85.63	36.63
F90–F99	Behavioral and emotional disorders with onset usually occurring in childhood and adolescence, unspecified mental disorder	45.19	38.36
F80–F89	Disorders of psychological development	66.20	38.50

*The bold values indicates that out of all diagnoses, only mental illnesses are considered in this article*.

*H4: Sample 1 and Sample 2 distributions are the same*.

Based on the current results, it is evident that there are differences in length of sick leave between different occupational category in both Sample 1 and Sample 2. The results are presented in Table 6.

**Table 6:** Pairwise comparisons: the average length of sick leave in the study sample of young adults aged 15–29 years, according to International Statistical Classification of Diseases and Related Health Problems, version 10, the Czech Republic, 2017.

**Sample 1–Sample 2**	**Sig**.	**Adj. Sig.^**a**^**
Plant and machine operators and assemblers–Professionals	0.986	1.000
Plant and machine operators and assemblers–Elementary occupations	0.281	1.000
Plant and machine operators and assemblers–Armed forces occupations	0.392	1.000
Plant and machine operators and assemblers–Technicians and associate professionals	0.082	1.000
Plant and machine operators and assemblers–Craft and related trades workers	0.066	1.000
Plant and machine operators and assemblers–Services and sales workers	0.013	0.580
**Plant and machine operators and assemblers–Clerical support workers**	**<0.001**	**0.000**
Plant and machine operators and assemblers–Skilled agricultural, forestry, and fishery workers	0.289	1.000
**Plant and machine operators and assemblers–Managers**	**<0.001**	**0.000**
Armed forces occupations–Professionals	0.853	1.000
Armed forces occupations–Elementary occupations	0.960	1.000
Armed forces occupations–Technicians and associate professionals	0.577	1.000
Armed forces occupations–Craft and related trades workers	0.463	1.000
Armed forces occupations–Clerical support workers	0.025	1.000
Armed forces occupations–Services and sales workers	0.059	1.000
Armed forces occupations–Skilled agricultural, forestry and fishery workers	0.546	1.000
Professionals–Managers	0.096	1.000
**Elementary occupations–Managers**	**<0.001**	**0.000**
Professionals–Elementary occupations	0.872	1.000
Professionals–Craft and related trades workers	0.884	1.000
Professionals–Technicians and associate professionals	0.858	1.000
Professionals–Clerical support workers	0.581	1.000
Professionals–Services and sales workers	0.654	1.000
Professionals–Skilled agricultural, forestry and fishery workers	0.794	1.000
Elementary occupations–Technicians and associate professionals	0.358	1.000
Elementary occupations–Services and sales workers	0.028	1.000
Elementary occupations–Clerical support workers	0.025	1.000
Elementary occupations–Skilled agricultural, forestry and fishery workers	0.555	1.000
Armed forces occupations–Managers	0.015	0.693
Elementary occupations–Craft and related trades workers	0.233	1.000
Craft and related trades workers–Clerical support workers	0.065	0.089
Craft and related trades workers–Services and sales workers	0.162	1.000
Technicians and associate professionals–Craft and related trades workers	0.925	1.000
Technicians and associate professionals–Services and sales workers	0.406	1.000
Technicians and associate professionals–Skilled agricultural, forestry and fishery workers	0.776	1.000
Technicians and associate professionals–Managers	0.040	1.000
Craft and related trades workers–Managers	0.026	0.765
Technicians and associate professionals–Clerical support workers	0.495	1.000
Craft and related trades workers–Skilled agricultural, forestry and fishery workers	0.793	1.000
Clerical support workers–Services and sales workers	0.776	1.000
Clerical support workers–Skilled agricultural, forestry and fishery workers	0.832	1.000
Services and sales workers–Skilled agricultural, forestry and fishery workers	0.762	1.000
Services and sales workers–Managers	0.105	1.000
Clerical support workers–Managers	0.092	1.000
Skilled agricultural, forestry and fishery workers–Managers	0.842	1.000

*The bold values indicates the most significant values*.

In our further analysis, we tested the null hypothesis that the Sample 1 and Sample 2 distributions are the same, whereas those samples present the work category. Kruskal–Wallis test provided very strong evidence of a difference (*p* < 0.001) between the mean ranks of at least one pair of groups. There was very strong evidence (*p* < 0.001, adjusted using the Bonferroni correction for multiple tests) of a difference between plant and machine operators and assemblers vs. clerical support workers, plant and machine operators and assemblers vs. managers, and elementary occupations vs. managers. There was no evidence of a difference between the other pairs.

## Discussion

Mental illnesses present serious health issues accompanied by various symptoms that negatively affect a person's behavior, emotional state, thoughts, and cognitive abilities. Many non-communicable diseases, leading to high social burdens, are associated with obesity, related pathologies, diabetes, high cholesterol, cardiovascular problems, mental diseases, etc. (32). Mental illnesses take many forms, from mild depression, anxiety to severe depression, and psychosis. Roberts & Grimes, Hewlett et al., and Patel et al. state that mental illnesses can have a short term but also a chronic form and affect the entire population of countries. The differentiation of age and gender of these mild as well as serious chronic mental diseases is proven (33–35). As stated in the previous studies of Song et al. and McDaid et al., the importance of solving the problem of mental illness of the population arises from health and economic aspects. Considering health, mental illnesses are often associated with a higher incidence of autoimmune diseases and worsen the treatment of these diseases or comorbidities (36, 37). People with severe mental illness live on average 20 years shorter, and general mental health problems shorten life expectancy by up to 7.5 years (32, 33). From an economic point of view, improving mental health has a positive impact on employment and productivity of the economy, it reduces social expenditures, because healthy people have a higher ability and preconditions to work efficiently.

Psychiatric patients have a greater risk of premature all-cause mortality than the general population. Epidemiological studies show that the life expectancy of patients with major psychiatric disorders is reduced by 7 to 24 years. Indeed, the cardiovascular disease (CVD) risk but also that of the related comorbidities of diabetes, stroke, and obesity has proven to be significantly increased in a multitude of psychiatric conditions, including depression, schizophrenia, bipolar disorder (BD), and anxiety disorder (34, 36).

Young people face stressful situations that influence their mental health. Mitchell et al. and Birn et al. confirmed that young adults are at high risk of developing mental disease and behavioral disorders as psychic of young people is not fully formed (2, 3). This might reduce their ability to work and therefore be wholly economically active and contribute to national GDP. Mental disorder in younger people might negatively influence their ability to find work, to gain good social status, and to become economically independent. If any kind of disease spread out in younger age, the economic burden of this person is higher as the years living with disability increase. In the case of mental illness, it is necessary to distinguish whether the illness has broken out as a result of substance abuse or is a different type of disease; therefore, mental disorders might be divided into two groups: mental diseases attributable to substance use (F10–F19) and mental diseases not attributable to substance use (F00–F09, F20–F99).

Generally, mental disorders occur in people who have long-term problems caused by social and psychological factors, etc. According to Liu et al., Wittink and Oosterhaven, and Duplaga, the great problem is people with limited health literacy, mostly people with lower education (14, 15, 17). It is necessary to create appropriate policy that deals with the problem of social inequality, different types of access to education and disadvantaged conditions in which a young person might grow up. People with higher education (such as managers, professionals, technicians, and associate professionals) are supposed to have better access to health care as they presumably care about their health and prevention more. This was confirmed in our pairwise analysis which shows that there are differences between managers (supposed to be higher educated, or care more about their healthcare) and plant and machine operators and assemblers (supposed to be opposite to managers).

On the contrary, people who have only primary education or are not educated at all do not usually care about their health as much as educated people. The social status of lower educated people might be worse and their health literacy is lower too. Therefore, as stated by Duplaga, Carod-Artal, and Zheng et al., healthcare of people, who do not care about their health, prevention, and healthier life, might be more complicated, longer, and more expensive (17–19). As the health system of Czech Republic is based on Bismarck model of health care, longer health problems related to longer sick leave will increase the expenses of state. According to many previous studies, it is supposed that the length of sick leave will be influenced by the social status and related work category (6, 8, 9).

First, we examined the differences in the length of sick leave in people with addiction, therefore people with sick leave related to diagnoses F10–F19. This assumption was not confirmed as the length of sick leave was higher in people with mental disorder attributable to other issues such as dementia in Alzheimer's disease, schizophrenia, and bipolar effective disorder, etc. As it is seen in Table 5, in case of both women and men, the average length of sick leave related to mental diseases caused by substance use is the fifth longest. In case of women, it is 43.3 days on average and in case of men, it is 44.86 days on average.

Previous studies confirm a relationship between lower education and a higher incidence of mental diseases (37–39). We supposed that people in occupational categories 9 (elementary occupations), 8 (plant and machine operators and assemblers), 7 (craft and related trades workers), and 6 (skilled agricultural, forestry, and fishery workers) have presumably lower education and will be more endangered by mental diseases related to addiction. Thus, we examined the existence of difference in median in the length of sick leave. However, this assumption was not confirmed. Therefore, we do not have enough evidence to confirm that length of sick leave, in people who have mental disease attributable to substance use, varies from one occupational category to another.

Considering mental disorders or behavioral issues that are not related to substance use (F00–F09 and F20–F99), our last assumption was that there is a difference in length of sick leave among different occupational categories. To make the analysis more detailed, we did the pairwise comparison to get to know exactly what kind of occupational categories are statistically significant from other groups.

There are many factors that influence the outbreak of mental disease and associated sick leave. Carod-Artal states that there are differences in morbidity related to mental diseases among men and women (18). It is confirmed by our findings, which show that the average number of days of sickness leave related to mental illness is higher for men compared to women. This is the important information for the policymakers. Furthermore, based on our results, considering only the mental disorders related to substances, there is no difference in working categories among young people with different levels of education. This may result from the fact that people who have some kinds of mental disorder attributable to addiction are presented in all working categories, or length of their sick leave is similar (40, 41). Thus, the addiction does not seem to be related to different occupational categories.

As our study shows, it is important to examine the differences in morbidity attributable to mental diseases and behavioral disorders. It is important to identify and monitor factors that can be a major cause of an outbreak of mental illness, especially in people with lower education. These people are more at risk of disease's outbreak because their health literacy is lower. The employment of a particular person can be the result of several factors. Thus, the key factor in employment is the educational level and type (42, 43).

Furthermore, nowadays, people are working under pressure, at work because of higher demands of employers (19). The sociocultural environment, political regulations, and public health strategies vary considerably among countries (44). Therefore, when setting health policies, it is necessary to perform the analyses that define vulnerable groups of the population in more detail taking into account also the differences in the occupational groups. In the case of mental illnesses that are not associated with the use of alcohol, tobacco, or illicit drugs, there are no differences in the duration of sick leave between people in individual occupational categories. On the contrary, the difference is evident in people with several mental diagnoses that are not attributable to substance use. Limitation of this study is the impossibility of comparison to other countries as our analysis is based on the data with specific structure. Data access to such a data in other countries may be limited by legislation. As the social system of countries might be different, this kind of data might exist in different structures or might not exist at all. Further analysis might be provided with the main aim to show which occupations are different from the others.

## Data Availability Statement

The data analyzed in this study is subject to the following licenses/restrictions: This research was supported by the Internal Grant Agency of FaME Tomas Bata University in Zlin: RVO/2022. Requests to access these datasets should be directed to gavurova@utb.cz.

## Ethics Statement

Ethical review and approval was not required for the study on human participants in accordance with the local legislation and institutional requirements. Written informed consent from the participants was not required to participate in this study in accordance with the national legislation and the institutional requirements.

## Author Contributions

BG: conceptualization, validation, investigation, and project administration. MT: methodology, formal analysis, and resources and data curation. BG and MT: writing—original draft preparation and writing—review and editing. All authors contributed to the article and approved the submitted version.

## Funding

This work was supported by Internal Grant Agency of FaME Tomas Bata University in Zlin: RO/2022: Use of corporate social responsibility in the hospitalisation sector in the Czech Republic and the Slovak Republic to increase efficiency and sustainability of the health systems and the Slovak Research and Development Agency: Contract No. APVV-17-0360: Multidimensional analysis of significant determinants of public procurement efficiency with emphasis on the application of Health Technology Assessment in the procurement preparation phase.

## Conflict of Interest

The authors declare that the research was conducted in the absence of any commercial or financial relationships that could be construed as a potential conflict of interest.

## Publisher's Note

All claims expressed in this article are solely those of the authors and do not necessarily represent those of their affiliated organizations, or those of the publisher, the editors and the reviewers. Any product that may be evaluated in this article, or claim that may be made by its manufacturer, is not guaranteed or endorsed by the publisher.
